# Metabolic engineering in woody plants: challenges, advances, and opportunities

**DOI:** 10.1007/s42994-021-00054-1

**Published:** 2021-06-23

**Authors:** Shu Yu, Cody S. Bekkering, Li Tian

**Affiliations:** Department of Plant Sciences, Mail Stop 3, University of California, Davis, CA 95616 USA

**Keywords:** Woody plant, Metabolic engineering, Bioeconomy, Sustainability

## Abstract

Woody plant species represent an invaluable reserve of biochemical diversity to which metabolic engineering can be applied to satisfy the need for commodity and specialty chemicals, pharmaceuticals, and renewable energy. Woody plants are particularly promising for this application due to their low input needs, high biomass, and immeasurable ecosystem services. However, existing challenges have hindered their widespread adoption in metabolic engineering efforts, such as long generation times, large and highly heterozygous genomes, and difficulties in transformation and regeneration. Recent advances in omics approaches, systems biology modeling, and plant transformation and regeneration methods provide effective approaches in overcoming these outstanding challenges. Promises brought by developments in this space are steadily opening the door to widespread metabolic engineering of woody plants to meet the global need for a wide range of sustainably sourced chemicals and materials.

## Introduction

Woody plants are resilient perennials defined by their characteristic woody stems and large root systems. Woody plants are a highly diverse group that is polyphyletic in origin and have both flowering and non-flowering members. Many species of woody plants have evolved since their origins around 380 million years ago, and they have since come to dominate various landscapes around the globe (Wilson et al. 2017). In contrast to herbaceous plants that can employ the strategy of escaping and avoiding stresses by dispersing their seeds (Chelli-Chaabouni 2014; Sade et al. 2018), perennial woody plants must tolerate stresses in the areas they occupy. Woody plants invest a large amount of energy in vegetative growth (e.g. producing wood) and have well-developed xylem, phloem, and root system that enable them to survive diverse environments and stress conditions. In this regard, some monocot species such as palm, though lacking secondary growth characteristics of true wood, have substantial metabolic investment in vegetative growth that affords them resilience comparable to true woody plants.

**Fig. 1:**
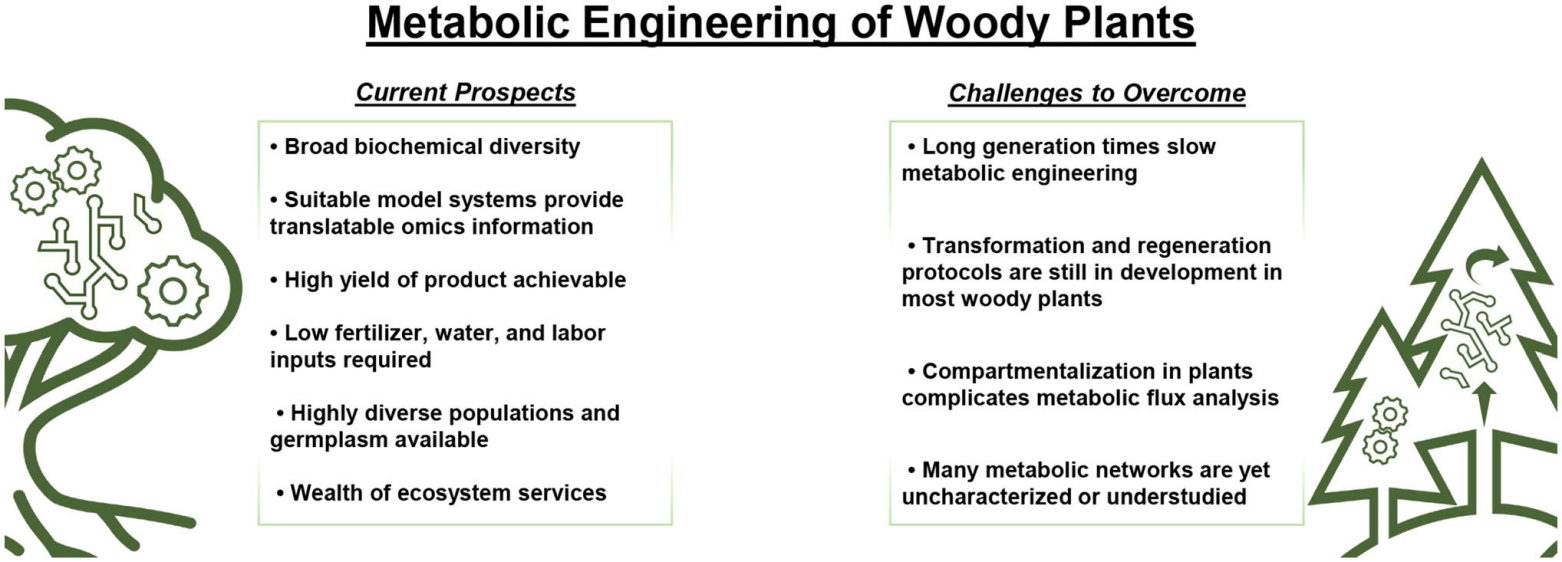
Prospects and current challenges of leveraging woody plants in metabolic engineering

Approximately 80% of the biomass on Earth is stored in forests comprised of diverse woody plant flora. This immense genetic diversity remaining in woody plants serves as a precious deposit of untapped metabolic pathways that provides raw materials for metabolic engineering. The value and novelty of woody plant metabolism are paralleled in scale by their immense size and growth rate, raising promise for their use as a sustainable platform for metabolic engineering (Fig. 1). Because of the large biomass available from woody plants after establishment, high yield of desirable bioproducts can be achieved—yields that can be further expanded in some systems through short rotation planting or coppicing schemes (Ragauskas et al. 2006). Perennial woody plant cultivation can operate with less inputs, such as fertilizer, water, and labor, and can also adapt to different environments including land too marginal for conventional food crops, lowering their impact on the global food system. Woody plants are favored for biofuel production from lignocellulosic biomass as it is not derived from food crops, also lowering their strain on the global food system (Bryant et al. 2020; Choi et al. 2020). Moreover, the ecological benefits of woody plants are also considerable—contributing habitat, shade, erosion protection, and soil carbon sequestration. This suite of advantages makes woody plants a sound platform for metabolic engineering in the sustainable bioeconomy of tomorrow.

Despite the broad advantages of leveraging woody plant systems for metabolic engineering, they present unique challenges also (Fig. 1). The life cycle of woody plants is often long and prohibitive to engineering approaches that require multiple generations. In addition, many valuable phytochemicals produced by woody plants are low in accumulation—with some being synthesized only in response to certain stimuli due to their function in defense (Burlacu et al. 2020; Oleszek et al. 2019). The physiological and genetic understanding of woody plant species is also generally exceeded by that of more intensively studied herbaceous plants (Burlacu et al. 2020). Finally, some woody plants are difficult to transform and regeneration rates of many woody plants following transformation remains low, adding difficulty to engineering approaches that require genetic transformation. Nonetheless, there is current progress in the study of woody plant biology that alleviates these inhibitions to metabolic engineering. In this review, we will focus on current advances in woody plant metabolic engineering that has met some of the pressing challenges to their implementation; in addition, promising trajectories will also be discussed that chart the course for woody plant metabolic engineering in the near future.

## Goals and progress of metabolic engineering of woody plants

### Improving the growth performance and disease resistance properties of woody plants

To mitigate biotic and abiotic stresses, woody plants produce a variety of specialized metabolites that belong to many chemical classes including (but not limited to) terpenoids, alkaloids, simple phenolics, coumarins, tannins, and lignins. There have been several examples of successful metabolic engineering efforts centered on the accumulation of such compounds in woody plants (Table 1). Proanthocyanidins (PAs) are phenolic metabolites that can induce plant defense mechanisms and protect plants from pathogens (Ullah et al. 2017; Wang et al. 2017). Overexpression of a transparent testa 2 (TT2)-like transcription factor *MYB115* in poplar enhanced the biosynthesis of PAs by positively regulating PA biosynthetic genes, which led to lessened disease symptoms when leaves were inoculated with the fungal pathogen *Dothiorella gregaria* (Wang et al. 2017). Resveratrol is a phenolic phytoalexin with protective roles for plants that is also an antioxidant associated with human health benefits. Metabolic engineering of piceid, a resveratrol glucoside, has been achieved in apple (*Malus domestica*) in two studies despite piceid not occurring naturally in apple (Kobayashi et al. 2000; Rühmann et al. 2006). In one example, expression of the *stilbene synthase* gene from grapevine (*Vitis vinifera*) in apple resulted in the accumulation of piceid in transgenic apple fruits (Rühmann et al. 2006). Although piceid has implications in phytopathology, these piceid-accumulating apple plants were not subjected to pathogen stress to test their disease resistant properties.

**Table 1:** Feasible targets for metabolic engineering within more commonly studied woody plant species

Plant family	Major metabolite classes for metabolic engineering	Physiological role	Engineering examples
Euphorbiaceae	Curcins	Biotic stress resistance	*Jatropha curcas*: Gu et al. (2015)
	Fatty acids/lipids	Carbon and energy reserve	*Jatropha curcas*: Maravi et al. (2016), Qu et al. (2012)
Myrtaceae	Monoterpenes	Defense	*Eucalyptus camaldulensis*: Ohara et al. (2010)
	Lignin	Structural support, defense	Hybrid eucalyptus: Sykes et al. (2015)
	Glycinebetaine	Abiotic stress tolerance	*Eucalyptus globulus*: Matsunaga et al. (2012)
Salicaceae	Lignin	Structural support, defense	Hybrid poplar: Baucher et al. (1996), Coleman et al. (2008), Huntley et al. (2003), Van Acker et al. (2014), Van Doorsselaere et al. (1995), Voelker et al. (2011)
	Carbohydrates	Carbon and energy reserve	Hybrid poplar: Coleman et al. (2007), Lee et al. (2009); White poplar: Park et al. (2004)
	Proanthocyanidins	Defense	Hybrid aspen: Mellway and Constabel (2009); Chinese white poplar: Wang et al. (2017), Yuan et al. (2012)
	Stilbenoids	Biotic stress resistance	White poplar: Giorcelli et al. (2004); Hybrid aspen: Seppänen et al. (2004)
	Glutamine	Nitrogen assimilation	Hybrid poplar: Jing et al. (2004)
	Glutathione	Defense	Hybrid poplar: Koprivova et al. (2002)
	Bioplastic (PHB)	Not natural in woody plants	Hybrid poplar: Dalton et al. (2011)
	Phenylpropenes	Attractant and defense	Hybrid aspen: Koeduka et al. (2013)
Actinidiaceae	Carotenoids	Photosynthesis, fruit color	Kiwifruit: Kim et al. (2010)
	Stilbenoids	Biotic stress resistance	Kiwifruit: Kobayashi et al. (2000)
Rosaceae	Stilbenoids	Biotic stress resistance	Apple: Rühmann et al. (2006), Szankowski et al. (2003)
	Polyamines	Abiotic stress tolerance	Common pear: Wen et al. (2008)
	Flavonoids	Defense and coloration	Apple: Rihani et al. (2017)
	Anthocyanins	Abiotic stress tolerance, fruit color	Apple: Espley et al. (2007)
Vitaceae	Stilbenoids	Biotic stress resistance	Grape: Coutos-Thévenot et al. (2001), Fan et al. (2008)
	Strigolactones	Growth regulation	Grape: Ren et al. (2020)
Rubiaceae	Alkaloids	Defense	Coffee: Ogita et al. (2004), Ogita et al. (2003)
Theaceae	Alkaloids	Defense	Tea tree: Mohanpuria et al. (2011a), Mohanpuria et al. (2011b)
Arecaceae	Bioplastic (PHB)	Not natural in woody plants	African oil palm: Parveez et al. (2015)
Pinaceae^†^	Lignin	Structural support, defense	Monterey pine: Wagner et al. (2009), Wagner et al. (2007), Wagner et al. (2011)
Rutaceae	Monoterpenes	Abiotic stress tolerance, insect attraction	Sweet orange: Rodríguez et al. (2018)
	Sesquiterpenes	Defense	Sweet orange: Alquézar et al. (2021)
	Carotenoids	Photosynthesis, fruit color	Sweet orange: Pons et al. (2014)
Asteraceae	Natural rubber	Defense	Guayule: Placido et al. (2019)
Juglandaceae	Ammonium	Nitrogen assimilation	Persian walnut: Liu, (2021)
Caricaceae	Stilbenoids	Biotic stress resistance	Papaya: Zhu et al. (2004)
Ebenaceae	Glycinebetaine	Abiotic stress tolerance	Japanese persimmon: Gao et al. (2000)
	Sorbitol	Abiotic stress tolerance, biotic stress resistance	Japanese persimmon: Gao et al. (2001)

Engineering successes for each target are shown

^†^Gymnosperm

Engineering of volatile organic compounds (VOCs) is similarly applied to the protection of woody plants from pests and pathogens by disrupting their interactions with the host plant (Pickett and Khan 2016). In one example, orange (*Citrus sinensis*) was engineered to produce less limonene (major VOC in oil glands of mature orange fruit) and instead accumulate increased monoterpene alcohols that share the common biosynthetic precursor geranyl pyrophosphate with limonene, increasing resistance to fungal pathogens that would otherwise lower yields and shelf life (Rodríguez et al. 2018). Overexpressing the *Arabidopsis *β*-caryophyllene synthase* gene in sweet orange leaves caused large emissions of a VOC β-caryophyllene. As β-caryophyllene functions as a repellent of *Diaphorina citri*, the transgenic orange plants showed reduced attraction to *D. citri*, an insect vector of the notorious crop disease “Huanglongbing”, in olfactometric behavioral assays and the choice behavioral test, demonstrating the potential of this approach for controlling Huanglongbing (Alquézar et al. 2021).

Phytohormones, despite their complex crosstalk, are also logical targets for metabolic engineering of woody plants due to their mediation of many processes including growth, stress tolerance, and phenology (Fenn and Giovannoni 2021). Auxins, gibberellins, brassinosteroids, cytokinins, jasmonic acid, salicylic acid, and ethylene can have their biosynthesis altered to change the biomass accumulation and architecture of woody plants to better suit agronomic usage or improve product quality (Dubouzet et al. 2013; Osakabe et al. 2011). Work in poplar has produced multiple successful examples—with hormone signaling being modified to produce fast growing, narrow trees amenable to usage in close stands (Mauriat and Moritz 2009; Nieminen et al. 2008). Ethylene biosynthesis, for example, has also been suppressed in apple to produce fruits with longer shelf life (Dandekar et al. 2004). However, complex crosstalk in hormone signaling can result in deleterious pleiotropic effects when hormone signaling is manipulated. Work on transgenic poplar demonstrates this, as lines overexpressing abscisic acid responsive element proteins displayed increased drought tolerance at the expense of biomass accumulation (Yu et al. 2019). Existing knowledge of hormonal signaling networks can be leveraged in woody plants to optimize growth and the value of the desired product while attempting to mitigate pleiotropic effects brought on by crosstalk. One such approach could be targeting specific transcription factors or receptors implicated in the hormone signaling rather than the hormone synthesis itself.

### Enhancing the nutritional quality of woody plant products and the production of pharmaceutical and specialty chemicals

Many tree fruits contain essential nutrients such as folate and ascorbic acid while also producing many nutraceutical compounds such as flavonoids, polyphenols, and carotenoids (Karasawa and Mohan 2018). Metabolic engineering of fruit nutritional quality and postharvest metabolic processes not only serves to add value to the consumable product, but also to improve health worldwide. There have been several initial successes in metabolic engineering of fruit nutritional quality in common fruit-bearing woody species (Table 1). Downregulating the expression of *MdMYB44*, a regulator of fruit acidity in apple, increased the accumulation of malate (a key flavor component) in transient transgenic apple calli (Jia et al. 2021). Sugar accumulation in apple was promoted by suppressing *aldose-6-phosphate reductase*, a key enzyme promoting sorbitol biosynthesis from glucose-6-phosphate in apple (Li et al. 2018). Apple engineered to constitutively overexpress a regulator of anthocyanin biosynthesis, the *Md*WRKY11 transcription factor, demonstrated high anthocyanin accumulation and red coloration in fruit flesh (Liu et al. 2019; Pons et al.^2014^). In another study, sweet orange was engineered with an RNAi construct to inhibit β-carotene hydroxylase activity, resulting in higher accumulation of β-carotene (a provitamin A carotenoid) in the fruit due to its reduced conversion to xanthophylls. This RNAi construct was transformed alongside of a *FLOWERING LOCUS T (FT)* overexpression cassette to considerably shorten the seed-to-seed time in the resulting lines (Pons et al. 2014).

Beyond nutritional compounds, woody plants synthesize a wealth of health-promoting medicinal compounds. Many such medicinal compounds are economic targets for metabolic engineering (Table 1) in addition to the outstanding examples of the anticancer drug paclitaxel from pacific yew tree (*Taxus brevifolia*) and the vaccine adjuvant QS-21 from *Quillaja saponaria*. Some chemical classes represented in woody plants used in herbalism are quinoline alkaloids (Such as those from the cinchona tree: cinchonine, cinchonidine, quinidine, and the antimalarial agent quinine){Dey, 2020 #281}, isoquinoline alkaloids (such as the anticancer and antiviral compound berberine from *Berberis* species), purine alkaloids (including caffeine from coffee trees, theophylline from tea plants and theobromine from cacao plants), benzophenones (the major bioactive compounds in mango leaves which are used to treat diabetes), and flavonoids (Dey et al. 2020; Neag et al. 2018; Zhang et al. 2019). Metabolic engineering is also central to the effort of increasing yields of specialty chemicals, such as components of fragrance and essential oils. For instance, with an aim of improving overall monoterpene production, the *Perilla frutescens limonene synthase* was overexpressed in *Eucalyptus camaldulensis* with a plastidic or cytosolic localization, resulting in over a threefold increase in limonene production alongside of increased accumulation of two other major monoterpenes, which are valued components of Eucalyptus essential oils (Ohara et al. 2010). On the other hand, “undesirable” metabolites can be reduced in woody plants through metabolic engineering. Transgenic coffee (*Coffea arabica* and *C. canephora*) expressing an RNAi construct suppressing *7-N-methylxanthine methyltransferase* showed up to a 50% reduction in caffeine content in embryonic tissues and plantlets—opening the door to naturally decaffeinated coffee (Ogita et al. 2004).

### Providing sources of renewable energy

Woody plants are a key second-generation biofuel—serving as a feedstock for both bioethanol and biodiesel production (Carriquiry et al. 2011). Two of the most desirable characteristics of woody plants for biofuel production are rapid growth rate and ability to be coppiced. Hybrid poplar (*Populus* spp.) and willow (*Salix* spp.) grow quickly and tolerate coppicing well, making them good targets of metabolic engineering for biofuel production. As high lignin content constrains the effectiveness of chemical pulping and of lignocellulosic ethanol production, ideal woody feedstocks for this process would also have optimal cellulose and lignin compositions and benefit from the redirection of metabolites away from lignin biosynthesis (Mahon and Mansfield 2019; Van Acker et al. 2014). The benefits of redirecting metabolic flux are potentially twofold: the energy demands of lignin biosynthesis can be lowered while simultaneously increasing the ease that valuable compounds can be harvested from the woody plant material. Suppression of enzymes responsible for lignin precursor production in transgenic poplar decreased the accumulation of insoluble lignin (Bjurhager et al. 2010; Coleman et al. 2008; Van Acker et al. 2014). In one study, this also increased the presence of soluble products including many phenylpropanoid glycosides (Coleman et al. 2008). In another study, this reduction of insoluble lignin fraction improved the ethanol yield of the fermented biomass, but with reduced biomass yield (Van Acker et al. 2014). As such, modifying lignin metabolism is a promising frontier to facilitate chemical production in woody plants, though the trade-off between the lignin reduction and sustained growth of the plants warrants further investigation.

Oil producing woody species such as palm (*Elaeis guineensis*) and *Jatropha curcas* are promising sources of biodiesel (Carriquiry et al. 2011). Current efforts are underway to engineer palm trees with enhanced production of oleic acid and with generally reduced saturated fatty acids (Rasid et al. 2020). In *J. curcas*, triacylglycerol (TAG) catabolism was suppressed by downregulating a lipase via RNAi, resulting in higher storage lipid accumulation in seeds (Kim et al. 2014). TAG accumulation in *J. curcas* seeds was also increased by overexpressing *Arabidopsis* *diacylglycerol acyltransferase 1*, a committed step in TAG biosynthesis (Maravi et al. 2016). Finally, RNAi cassettes have also been leveraged in *J. curcas* to stably lower the expression of toxic curcin proteins in the seeds, addressing the safety concerns of jatropha processing (Gu et al. 2015). These approaches highlight possibilities in long-lived oil producing species as transformation and regeneration tools become more standardized and widely implemented.

## Development of model systems and omics and systems biology tools to expedite metabolic engineering of woody plants

### Utility of model systems and omics approaches in metabolic engineering of woody plants

With the availability of various genetic, genomic, and biochemical tools, poplar have emerged as suitable model systems for woody plant biology, particularly for important tree-specific traits such as crown size, trunk diameter, and wood density. Multiple annotated, high coverage genome sequences exist for *Populus* species, such as *P. trichocarpa*, *P. deltoides*, *P. tremula* × *alba* hybrid, *P. euphratica*, and *P. tremula* (Osakabe et al. 2016; Tuskan et al. 2018). A subset of these genomes and a standardized gene expression atlas of *P*. *trichocarpa* are also integrated into the Phytozome platform for easier exploration (Goodstein et al. 2012). The availability of reference genomes expediate the sequencing-based methylome analysis to study the epigenetic control of metabolism. For example, high coverage genome-wide cytosine methylation maps and transcriptomic profiling of *P. trichocarpa* stems in different developmental stages were generated and compared to understand the roles of methylation in wood formation (Zhang et al. 2020). Whole-genome resequencing of outcrossing woody plants including poplar can be leveraged to map agronomically important loci using the recently developed OutcrossSeq method (Chen et al. 2021). In this method, a pair of founder parents are sequenced with high resolution, with progeny individuals from the founder cross sequenced at low coverage to produce haplotypes suitable for genetic mapping using fastGWA (Jiang et al. 2019). OutcrossSeq was validated on a biparental cross in walnut (*Juglans regia*) and can be extended to metabolic phenotypes that are potential targets of metabolic engineering not only in walnut, but also in other highly heterozygous woody plant species for which a reference genome exists (Chen et al. 2021).

Phenotypic data are critical when using the growing collection of woody plant genomic information to unravel the function of structural and regulatory genes for metabolic engineering. Plant phenomics is increasingly capable of meeting this need for phenotypic information because of recent advances in computing power and applications of computer vision. Classical image-based methods are useful for phenotyping smaller, more easily managed woody plant species such as grape (Underhill et al. 2020) and *Camellia sinensis* (Hazra et al. 2018). Image-based approaches for analyzing canopy growth from ground level are also available for tree species. A maintained online repository of these image-based tools can be found at Quantitative-plant.org (Lobet et al. 2013). Camera-equipped unmanned aerial vehicles (UAVs) can capture phenotypes otherwise inaccessible from the ground. Use of infrared cameras expands the collection to phenotypes available to researchers to spectral indices that can serve as suitable proxies for chemical composition. These include the modified simple ratio for chlorophyll content (Wu et al. 2008), anthocyanin reflectance index for anthocyanin pigment compounds (Gitelson et al. 2001), and the photochemical reflectance index for light absorbing compounds such as xanthophylls and carotenoids (Peñuelas et al. 2011). A clear limitation for implementing common phenomics tools into metabolic engineering efforts is their restriction to visible phenotypes. Subtle changes in metabolic flux are unlikely to result in clear changes in color or morphology. However, the association of a clear morphological or phenological phenotype with a controlled metabolic change raises the possibility of leveraging phenomics-based approaches as a fast, low-cost way to screen whole plots of woody plants. Ongoing work in this area will expand the availability and validation of spectral indices that are correlated with chemical content—thus affording more utility to the UAV-based systems that are already an asset to woody plant management.

Knowledge of both genetic and phenotypic data for species such as poplar opens the door to functional genomics in woody plants, which informs metabolic engineering. Genetic polymorphisms including common and rare variants have been identified in accessions covering wide latitudinal range of *Populus* species (Geraldes et al. 2013; Piot et al. 2020). Genome-wide association studies (GWAS) have taken advantage of these accessions to understand quantitative traits and, combined with the metabolic and phenomic data, to study the genetic regulation of metabolites (Geraldes et al. 2013; Piot et al. 2020). Systems biology approaches have integrated genetic and multi-omics information to uncover key players such as biosynthetic genes and transcription factors involved in the regulatory network (Myburg et al. 2019). In one application, integrated omics analysis was applied to transgenic poplar lines in which 21 lignin pathway genes were perturbed to predict traits associated with wood formation for improved lignin and wood properties in tree species (Wang et al. 2018a). Extensions of this work could be found in other metabolites linked to plant performance such as phenolics, carotenoids, and lipids.

Metabolic flux analysis examines intracellular carbon flows and constitutes an integral component of systems biology modeling. The long-distance transportation of metabolites through the extended vascular system of woody plants makes simulation of whole-plant metabolism using single cell models more challenging. Thus, modifications to the pipelines established in herbaceous model plants is needed (Junker 2014; Zhang et al. 2018). Work in poplar has nonetheless taken advantage of metabolic flux analysis to unpack the metabolic response to low nitrogen, elucidating a higher flux through the tricarboxylic acid (TCA) cycle and lower flux into nitrogen storage proteins under reduced nitrogen (Zhang et al. 2018). In another interesting example in woody plants, metabolic flux in transgenic poplar with suppressed isoprene synthase was compared to wild-type plants. This work found substantially reduced isoprene emission and increases in metabolic investment into chlorophyll and carotenoid pigments in the transgenic plants (Ghirardo et al. 2014). Overall, this plethora of genetic and omics information in *Populus* species enables exploratory studies of woody plant functional genomics even prior to experimental design in other woody systems. These pipelines and their findings can thus be transferred from woody model systems to other less-studied woody plants.

### Application of gene editing tools propelled by advances in transformation and regeneration of woody plants

Metabolic engineering often involves manipulating gene expression and function in plants. As a widely used gene editing technology, clustered regularly interspaced short palindromic repeats/CRISPR-associated protein 9 (CRISPR-Cas9) endonuclease systems have been used in woody plants such as poplar (*Populus tomentosa* Carr.), mandarin orange (*Citrus reticulata*), apple, pear (*Pyrus communis*), and grape (Charrier et al. 2019; Fan et al. 2015; Osakabe et al. 2016; Sandhya et al. 2020; Wang et al. 2018b). A great advantage of (modified) CRISPR-Cas9 is that multiple genes and gene homoeologs (in polyploid species) can be edited simultaneously in woody plants where pyramiding multiple gene mutations/overexpression by crossing is often not feasible (Armario Najera et al. 2019). In addition, homozygous mutations are possible as early as the T_0_ generation, and the otherwise low likelihood of this occurring can be improved using two or more guide RNAs targeting the same gene—an approach that has been successfully applied in multiple woody plants including poplar and grape (Fan et al. 2015; Wang et al. 2018b). Recently, Cas9-derived base editors have been developed and validated in poplar hybrids, which further expand the usage of CRISPR-Cas9 system in high efficient and precise genome editing in woody plants (Li et al. 2021). The expansion of CRISPR-Cas9 application to woody plants allows also more thorough exploration of biosynthetic enzymes and their regulation, which are currently understudied.

Despite the promise of gene editing technology, the transformation and regeneration of woody plants are challenging which poses a bottleneck to the application of CRISPR-Cas9 technologies in woody plants. To this end, methods for transforming and regenerating otherwise recalcitrant woody species are steadily being developed. Agroinfiltration into aerial tissues has been used successfully to derive stable transformants in woody plants such as poplar (Stettler et al. 1996), *Eucalyptus spp.* (Spokevicius et al. 2005; Tournier et al. 2003), oil palm (Masani et al. 2018; Yarra et al. 2019), grape (Das et al. 2002; Li et al. 2008), *Pinus taeda* (Wenck et al. 1999), and others. Infiltration of *Agrobacterium* into seedling tissues or extracted embryos has also proven successful in species such as peach and eastern white pine (*Pinus strobus*) (Ricci et al. 2020; Tang et al. 2007). Poor regeneration frequencies from aerial tissue remain an issue, however, for many woody plant species, and the success in regenerating woody plants from leaves has been found to be dependent on the genetic background in a few species (Stettler et al. 1996; Tournier et al. 2003).

Besides aerial tissues, hairy root tissue has proven to be an asset for genetic transformation of woody plants. Inoculating wounded plants with *Agrobacterium rhizogenes* induces the formation of hairy roots that can be cultured and regenerated into viable plants (Tepfer 1990). In purple willow (*Salix purpurea*), transformation efficiencies using hairy root culture far exceeded aerial tissues, showing efficiencies over 80% alongside of higher regeneration rates (Gomes et al. 2019). Highly effective transformation and regeneration protocols using hairy roots induced by *A. rhizogenes* have been demonstrated in several systems including *Eucalyptus* (Plasencia et al. 2016), *Poplar* (Yoshida et al. 2015), and *Camellia sinensis* (Alagarsamy et al. 2018). The efficacy of this method combined with the ability to introduce multi-gene cassettes using *A. rhizogenes* will make it a staple in woody plant genetic and metabolic engineering.

To promote regeneration of woody plant tissues, plant developmental regulators, which are transcription factors involved in reprograming cell fates, makes the metabolic engineering possible in some difficult to regenerate woody plants (Ikeuchi et al. 2019). Overexpressing developmental genes such as *GROWTH-REGULATING FACTOR 4 (GRF4)*, *GRF-INTERACTING FACTOR 1 (GIF1)*, *LEAFY COTYLEDON 1 (LEC1)*, *WUSCHEL*, and *BABY BOOM* (*BBM*) successfully improved the regeneration of *Arabidopsis* (Boutilier et al. 2002; Lotan et al. 1998; Luo and Palmgren 2021; Zuo et al. 2002). Similar strategies have also been tested in woody plants through ectopic expression of *GRF*–*GIF* chimeras in citrus (Debernardi et al. 2020); *LEC2* and *BBM* in *Theobroma cacao* (Florez et al. 2015; Shires et al. 2017); and *BBM* in Poplar (Deng et al. 2009), among others. Manipulating developmental regulators should be done with caution; however, as they may affect the development of newly generated transgenic plants. In *T. cacao*, in spite of the significant improvement in early stages of embryogenesis, homologous overexpression of *BBM* in cotyledons led to the occurrence of abnormal cotyledon development (Florez et al. 2015). An interesting new development in multiple dicotyledonous plant species including grape is that combinations of developmental regulators can be expressed through de novo meristem induction thus bypassing the need of in vitro tissue culture (Maher et al. 2020). These strategies need to be further tested and improved upon in additional woody plant species to explore and mitigate broader developmental issues caused by manipulating growth regulators for improved regeneration.

In addition to the aforementioned developmental regulators, additional factors have been applied to reduce the long juvenile period and promote flowering in woody plants to expedite breeding. For instance, the overexpression of *BpMADS4* successfully reduced the juvenile period and promoted early flowering in apple (Flachowsky et al. 2007). Ectopic expression of *FT* from many donor species has reduced the generation time woody species including plum (*Prunus domestica*) (Petri et al. 2018; Srinivasan et al. 2012), *Eucalyptus grandis* × *E. urophylla* hybrids (Klocko et al. 2016), and sweet orange (Pons et al. 2014). Overexpression of *Arabidopsis APETALA1* (*AP1*) shortened the generation time to one year in sweet orange and citrange (a hybrid of sweet orange and trifoliate orange; *Citrus sinensis* × *Poncirus trifoliata*). In the case of citrange, the transgenic plants were used in re-transformation to test gene stacking; the reporter genes were stably expressed in the re-transformant and were evaluated as early as one year (Cervera et al. 2009). The success of these approaches at substantially lowering generation time in woody species raises promise for introducing useful traits such as disease resistance from wild species or other elite lines.

## Metabolic engineering of agronomically and economically viable woody plants is enhanced by modern breeding tools

Traditional crossing and evaluation methods remain indispensable for facilitating metabolic engineering of woody plants because of their ability to combine locally adapted traits into engineered lines expressing a particular suite of biosynthetic and regulatory genes. Woody plant breeding is naturally hindered by the long time period before reproductive maturity and before meaningful phenotypic evaluation can occur. Strategies common in annual plant breeding such as “speed breeding” (Watson et al. 2018) and usage of off-season nurseries are not generally viable for woody species. An expanding toolkit of computational and engineering tools ameliorate this constraint and collectively speed up the process of traditional breeding in long-lived woody plants. Phenomic and genomic data for woody plants are central to this effort by collectively facilitating quantitative genetic approaches to breeding including genomic selection (GS)—that is, selection on whole genomes in highly heterogeneous populations (Lebedev et al. 2020). In this approach, genome-estimated breeding values (GEBVs) are estimated from genetic data (e.g. SNP arrays, whole genome sequences) and known phenotypes using statistical models such as Bayes Least Absolute Shrinkage and Selection Operator (Bayes LASSO) (Park and Casella 2008) and Ridge Regression Best Linear Unbiased Predictor (RR-BLUP) (Endelman 2011). Approaches using either family of models are increasingly relevant to breeding and can be applied effectively to woody plants.

Growth traits such as height, wood density, bark thickness, stem straightness, and circumference have been predicted in woody plant progeny with suitable accuracy using GS (de Moraes et al. 2018; Li et al. 2019b; Thistlethwaite et al. 2020). In addition, GS has also been used to predict yield in significant agricultural crops such as macadamia (*Macadamia integrifolia* × *tetraphylla*) and passion fruit (*Passiflora edulis*) (O’Connor et al. 2018; Viana et al. 2017). Finally, black spot resistance in pear progeny has been adequately predicted using multiple Bayesian GS models (Iwata et al. 2013). GS can also be applied to metabolic phenotypes, which has been demonstrated for anthocyanin content in maize and glucosinolate content in *Brassica napus* (Chatham and Juvik 2020; Werner et al. 2018). Using genome-wide information to predict metabolite profiles in woody plants has been relatively limited. There has been some application of GS to flavor components of grape and pear (Iwata et al. 2013; Viana et al. 2016). Recent work has also been conducted to predict rubber production in the progeny of rubber trees (*Hevea brasiliensis*) (Cros et al. 2019). Future applications of GS to woody species can be used in functional genomics to identify target traits and breeding schemes to optimize accumulation of useful metabolites or to increase flux through a metabolic pathway that can be leveraged in engineering efforts.

## Metabolic engineering beyond woody plants—producing woody plant metabolites in complementary systems

To obtain valuable metabolites naturally produced by woody plants, metabolic pathways can also be reconstructed in complementary biological systems. Microorganisms are suitable for this purpose because of their small genome, simple compartments, fast growth rate, and easily controllable growth conditions. For example, the synthesis of hydroxytyrosol, a valuable nutraceutical and preservative from olive, was achieved in *E. coli* through the heterologous expression of the hydroxytyrosol pathway in tandem with tyrosine overproduction (Trantas et al. 2019). In another example, the production of caffeine has also been achieved by inserting *xanthosine methyltransferase* from *Coffea arabica* and *caffeine synthase* from *Camellia sinensis* into yeast strains that were fed exogenous xanthosine (Jin et al. 2014). However, lack of the native biochemical and regulatory network in the microbial systems can result in metabolic imbalance, accumulated intermediates, and toxic compounds that lead to the failure of metabolic engineering in microbial systems. Moreover, maintaining a large, sterile manufacturing platform requires often prohibitive amounts of labor and materials that complicate scaling.

Using plants as a complementary system for metabolite production is often fruitful, as the availability of the existing upstream substrates with the native gene regulatory network circumvent issues found in microbial systems. The production of woody plant metabolite taxadiene (the first committed intermediate in the synthesis of taxol/paclitaxel, an anticancer drug) was boosted in multiple complementary plant systems that produce carotenoids such as tomato (*Solanum lycopersicum*) (Cha et al. 2012; Kovacs et al. 2007; Li et al. 2015). Taxadiene and carotenoids use the common biosynthetic precursor geranylgeranyl diphosphate (GGPP). By expressing the *taxadiene synthase* in a yellow flesh tomato with low accumulation of carotenoids due to lacking a functional phytoene synthase (the rate limiting enzyme for carotenoid biosynthesis), metabolic flux was redirected from carotenoid to taxadiene biosynthesis and resulted in high amounts of taxadiene in leaves and fruits of the transgenic plants (Besumbes et al. 2004; Kovacs et al. 2007). In another study, the accumulation of biosynthetic intermediates of taxol, taxadiene, and taxadiene-5α-ol, was significantly improved in *Nicotiana benthamiana* leaves by enriching the isoprenoid precursor for taxol biosynthesis and engineering enzymes for taxol biosynthesis in the same compartment (Li et al. 2019a).

The success above highlights the possibilities when many components of a biosynthetic pathway are known. However, de novo biosynthesis of woody plant metabolites in complementary systems can be challenging when the biosynthetic pathway is not fully elucidated (such as QS-21 and quinine). To this end, biosynthetic precursors and their derivatives can be fed to the complementary systems to produce downstream products (Cravens et al. 2019). Alternatively, pathway intermediates produced by the complementary systems can be extracted and used as substrates for in vitro reactions that produce the target woody plant metabolites (Birchfield and McIntosh 2020; Cravens et al. 2019). Metabolites that function in preserving the health of plant tissues or otherwise maintaining physiological roles are not suitable for production in heterologous plant expression systems, as extraction removes them from the context in which they are the most valuable. This applies to an array of compounds involved in flower color, lignin production, water homeostasis, and signaling. Nonetheless, heterologous systems remain useful for engineering efforts in which a purified final product is the central aim.

## Concluding remarks and future perspectives

Pivotal discoveries in herbaceous model systems such as *Arabidopsis*, tobacco, and tomato can help chart the course for research in woody species. Ongoing work in model systems is optimizing the delivery of multiple genes concurrently—which raises the possibility of circumventing multiple regeneration events for recalcitrant woody plants. Gene Assembly in *Agrobacterium* by Nucleic Acid Transfer using Recombinase Technology (GA*A*NTRY) uses multiple unidirectional site-specific recombinases to stack multiple genes in vivo. The GA*A*NTRY ArPORT1 strain of *A. rhizogenes* was demonstrated to be effective at stacking up to ten genes in rice (Hathwaik et al. 2021). As *A. rhizogenes* has proven effective at transforming woody plants (Gomes et al. 2019), the GA*A*NTRY system could prove useful for reconstituting metabolic pathways into woody plant chasses. Plant Artificial Chromosomes (PACs) are stable in plant cells and multiple genes on artificial chromosomes can be stably expressed. PACs have been the subject of research in non-woody plants such as barley, maize, and *Arabidopsis*, and promise remains for implementing PACs into woody plant species with established transformation and regeneration methods (Yu et al. 2016). Delivery of CRISPR-Cas9 systems is also being expanded, with viral delivery systems already developed and nanoparticle or nanotube delivery on the horizon (Demirer et al. 2021), facilitating the metabolic engineering effort in woody plants.

Though gene regulatory networks have been extensively explored in herbaceous plants, considerable work remains to gain a more complete understanding of such regulatory networks in woody plants. The task of unraveling these networks in woody plant species is complicated by the diversity of tissue types present as well as the maturation time needed for such gene networks to emerge and be adequately explored. Poplar serves as a suitable woody model plant from which findings can be translated. Methods and knowledge developed in poplar can prove instrumental in exploring other woody plant systems to bolster our knowledge of their metabolic networks and to translate suitable approaches for metabolic engineering. Conquering the bottlenecks of transformation and regeneration in non-model woody plants will shorten the design-build-test-learn cycles in gene editing and transgene insertion to streamline their usage in metabolic engineering as well. Finally, a large amount of untapped diversity exists in domesticated cultivars across broad geographic ranges and non-domesticated genotypes. Comparative genomics can take advantage of the highly diverse genomes of woody species around the globe not only for breeding, but also to study the evolution of genes and gene families to facilitate gene mining for metabolic engineering (Tuskan et al. 2018).

As woody plants are understudied, there is room to integrate current genetic and metabolic engineering strategies developed in other plant and microbial systems. Implementation of these strategies is finding increasing success in more studied woody plant systems such as poplar and citrus. Translating these tools into more woody plant systems not only leverages different native metabolic networks, but also expands the collection of ecosystem services available to the site of interest due to the broader species selection. These ecosystem services brought by woody plants are unique among plant chemical factories and include habitat, carbon sequestration, erosion reduction, and water retention. These ecosystem services can be viewed as the key benefits of a sustainable bioeconomy anchored in the used of long-lived woody plants. Low resource and labor demand further complement the vast ecosystem services of woody plant platforms. Metabolic engineering expedites our capability to simultaneously capitalize on the rich biochemical diversity of woody plants in tandem with the suite of environmental benefits associated with their usage. The transition from an oil-based economy to a sustainable bio-based economy will be facilitated by advances in this space.
